# Epidemiological and evolutionary considerations of SARS-CoV-2 vaccine dosing regimes

**DOI:** 10.1126/science.abg8663

**Published:** 2021-03-09

**Authors:** Chadi M. Saad-Roy, Sinead E. Morris, C. Jessica E. Metcalf, Michael J. Mina, Rachel E. Baker, Jeremy Farrar, Edward C. Holmes, Oliver G. Pybus, Andrea L. Graham, Simon A. Levin, Bryan T. Grenfell, Caroline E. Wagner

**Affiliations:** 1 Lewis-Sigler Institute for Integrative Genomics, Princeton University, Princeton, NJ 08540, USA.; 2 Department of Pathology and Cell Biology, Columbia University Medical Center, New York, NY 10032, USA.; 3 Department of Ecology and Evolutionary Biology, Princeton University, Princeton, NJ 08544, USA.; 4 Princeton School of Public and International Affairs, Princeton University, Princeton, NJ 08544, USA.; 5 Departments of Epidemiology and Immunology and Infectious Diseases, Harvard School of Public Health, Boston, MA 02115, USA.; 6 High Meadows Environmental Institute, Princeton University, Princeton, NJ 08544, USA.; 7 The Wellcome Trust, London NW1 2BE, UK.; 8 Marie Bashir Institute for Infectious Diseases and Biosecurity, School of Life and Environmental Sciences, and School of Medical Sciences, The University of Sydney, Sydney, NSW 2006, Australia.; 9 Department of Zoology, University of Oxford, Oxford OX1 3SZ, UK.; 10 Fogarty International Center, National Institutes of Health, Bethesda, MD 20892, USA.; 11 Department of Bioengineering, McGill University, Montreal, QC H3A 0C3, Canada.

## Abstract

Given vaccine dose shortages and logistical challenges, various deployment strategies are being proposed to increase population immunity levels to severe acute respiratory syndrome coronavirus 2 (SARS-CoV-2). Two critical issues arise: How timing of delivery of the second dose will affect infection dynamics and how it will affect prospects for the evolution of viral immune escape via a buildup of partially immune individuals. Both hinge on the robustness of the immune response elicited by a single dose as compared with natural and two-dose immunity. Building on an existing immuno-epidemiological model, we find that in the short term, focusing on one dose generally decreases infections, but that longer-term outcomes depend on this relative immune robustness. We then explore three scenarios of selection and find that a one-dose policy may increase the potential for antigenic evolution under certain conditions of partial population immunity. We highlight the critical need to test viral loads and quantify immune responses after one vaccine dose and to ramp up vaccination efforts globally.

## One dose or two?

For two-dose vaccines against severe acute respiratory syndrome coronavirus 2, some jurisdictions have decided to delay the second dose to rapidly get the vaccine into more people. The consequences of deviating from manufacturer-prescribed dosing regimens are unknown but will depend on the strength of immune responses to the vaccines. Saad-Roy *et al.* took a modeling approach to tackling the inevitable uncertainties facing vaccine rollout. The authors found that although one-dose strategies generally reduce infections in the short term, in the long term, the outcome depends on immune robustness. A one-dose strategy may increase the potential for antigenic evolution if immune responses are suboptimal and the virus continues to replicate in some vaccinated people, potentially leading to immune-escape mutations. It is critical to gather serological data from vaccinated people and, to avoid negative outcomes, to ramp up vaccination efforts worldwide.

*Science*, this issue p. 363

## Abstract (structured)

**INTRODUCTION:**

As the severe acute respiratory syndrome coronavirus 2 (SARS-CoV-2) pandemic continues, the deployment of safe and effective vaccines presents a key intervention for mitigating disease severity and spread. Numerous logistical challenges and shortages have emerged alongside the international distribution of approved vaccines. In response, several countries have chosen to delay the second dose in an effort to increase the number of individuals receiving at least one dose. A key question then becomes how the timing of delivery of the second dose will affect future epidemiological and evolutionary outcomes.

**RATIONALE:**

We build on an existing immuno-epidemiological framework that assumes that, without vaccination, individual immunity after recovery from primary infection may eventually wane, leading to (a potentially reduced) susceptibility to secondary infections. To explore epidemiological outcomes, we extend the model to incorporate two vaccinated classes, corresponding to individuals who have received either one dose or two doses of a SARS-CoV-2 vaccine. As with natural immunity, we allow for one- or two-dose vaccinal immunity to wane, and we consider a continuous spectrum for the interdose period between vaccines. To reflect the increase in available doses resulting from a delayed second dose, we model the rate of administration of the first dose as an increasing function of the interdose period. We then consider evolutionary outcomes by coupling this framework to a simple phylodynamic model for potential viral adaptation under different evolutionary scenarios, each with its own assumptions regarding viral abundance and within-host selection for the different partially susceptible classes.

**RESULTS:**

We find that delaying second vaccine doses reduces COVID-19 infections in the short term by increasing the proportion of immune individuals. In the longer term, however, both the infection burden and the relative potential for viral adaptation are highly dependent on the robustness of natural or vaccinal immune responses. Notably, we find that even if immunity conferred by a single vaccine dose is poor, starting with a one-dose policy early on to increase the number of individuals immunized and then switching to the manufacturer-recommended two-dose regime as vaccine capacity increases can mitigate potential negative longer-term epidemiological and evolutionary outcomes. This mitigation can also be achieved by ramping up overall vaccination rates as availability improves.

**CONCLUSION:**

The deployment of SARS-CoV-2 vaccines will strongly shape postpandemic epidemiological trajectories and characteristics of accumulated population immunity. Our models show that the combination of different vaccine dosing regimes and variations in the robustness of natural and vaccinal immunity may result in a wide range of potential epidemiological and evolutionary outcomes in the medium term. It is therefore imperative to determine the strength and duration of clinical protection and transmission-blocking immunity through careful clinical evaluations in order to enforce sound public policies. In places where vaccine deployment is delayed and vaccination rates are low, our results stress the subsequent negative epidemiological and evolutionary impacts that may emerge. Particularly because these consequences (for example, the evolution of new variants) could emerge as global problems, there is an urgent need for global equity in vaccine distribution and deployment.

## Teaser

A one-dose vaccine policy may increase the potential for viral antigenic evolution in some scenarios.

## Graphical abstract

**Figure:**
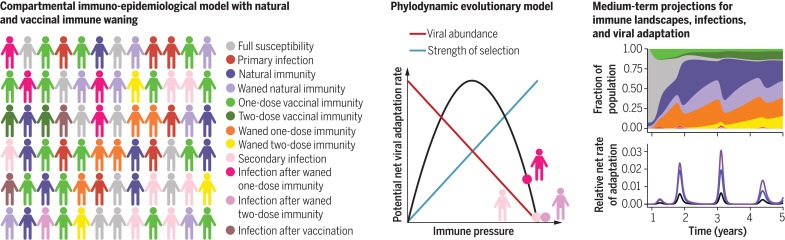
The relative robustness of one- or two-dose vaccinal immunity and natural immunity shape future epidemiological and evolutionary outcomes for SARS-CoV-2. An immuno-epidemiological model (left) coupled with a phylodynamic model (middle) is used to explore projections for COVID-19 infection burden and immune landscapes (top right) and potential rates of SARS-CoV-2 viral adaptation (bottom right) in the medium term. The accompanying online interactive application (http://grenfelllab.princeton.edu/sarscov2vaccine) can be used to explore these projections for a broad range of model parameters.

As the severe acute respiratory syndrome coronavirus 2 (SARS-CoV-2) betacoronavirus pandemic continues, the deployment of safe and effective vaccines presents a key intervention for mitigating disease severity and spread and eventually relaxing nonpharmaceutical interventions (NPIs). At the time of writing, 11 vaccines have been approved by at least one country (*1*). We focus mainly on the vaccines from Pfizer/BioNTech, Moderna, and Oxford/AstraZeneca. The first two elicit adaptive immunity against SARS-CoV-2 in response to the introduction of mRNA molecules that encode the spike protein of SARS-CoV-2 (*2*) and appear to offer greater than 95% [Pfizer/BioNTech (*3*), approved in 60 countries] and 94% [Moderna (*2*), approved in 38 countries] protection against symptomatic COVID-19. Both of these mRNA vaccines were tested in clinical trials according to a two-dose regime with dose spacing of 21 and 28 days for the Pfizer/BioNTech and Moderna platforms, respectively. The Oxford/AstraZeneca vaccine uses a nonreplicating adenovirus vector and has also been tested in clinical trials according to a two-dose regime with a target 28-day interdose period (although for logistical reasons some trial participants received their second dose after a delay of at least 12 weeks). Clinical trials indicated 62 to 90% efficacy for this vaccine depending on the specific dose administered (*4*). Although we base our parameter choices and modeling assumptions on these three vaccines, our results are generalizable across platforms.

As these vaccines have been distributed internationally, several countries, including the UK (*5*) and Canada (*6*), have chosen to delay the second dose in an effort to increase the number of individuals receiving at least one dose or in response to logistical constraints (*7*). Although a number of participants dropped out after a single dose of the vaccine in the Pfizer/BioNTech and Moderna trials, these studies were not designed to assess vaccine efficacy under such circumstances, and Pfizer has stated that there is no evidence that vaccine protection from a single dose extends beyond 21 days (*5*), although other data paint a more optimistic picture (*8*, *9*). The Oxford/AstraZeneca clinical trials did include different dose spacings, and limited evidence suggests that longer intervals (2 to 3 months) did not affect, and may even have improved, vaccine efficacy (*4*, *5*). Ultimately, the consequences of deviating from manufacturer-prescribed dosing regimes at the population scale remain unknown but will hinge on immune responses.

Although there has been considerable progress in quantifying host immune responses after infection (*10*–*12*), substantial uncertainty regarding the strength and duration of both natural and vaccinal SARS-CoV-2 immunity remains. Previous work suggests that these factors will play a central role in shaping the future dynamics of COVID-19 cases (*13*). Future cases also create an environment for the selection of novel variants [e.g., (*14*–*16*)]. Of particular concern is the possibility of antigenic drift [e.g., for influenza (*17*) and for the seasonal human coronavirus 229E (*18*)] via immune escape from natural or vaccinal immunity. For example, immune escape might be especially important if vaccinal immunity elicited after the complete two-dose regime is highly protective, whereas a single vaccine dose provides less effective immunity. Consequently, the longer-term epidemiological and evolutionary implications of these different SARS-CoV-2 vaccine dosing regimes are not yet clear; the immediate need for effective mass vaccination makes understanding them critical for informing policy (*19*).

Here, we explore these epidemiological and evolutionary considerations with an extension of a recent immuno-epidemiological model for SARS-CoV-2 dynamics (*13*), depicted schematically in Fig. 1. Without vaccination, our model reduces to the susceptible-infected-recovered(-susceptible) [SIR(S)] model (*13*, *20*), where individual immunity after recovery from primary infection may eventually wane at rate δ, leading to potentially reduced susceptibility to secondary infections, denoted by the fraction ϵ relative to a baseline level of unity. This parameter ϵ is thus related to the (transmission-blocking) strength of immunity and titrates between the SIR (lifetime immunity, ϵ = 0) and SIRS (hosts regain complete susceptibility, ϵ = 1) paradigms. Quantifying ϵ is challenging because it requires measuring reinfection rates after the waning of immunity. Some studies have made substantial progress in this direction (*11*, *12*); however, uncertainties remain, particularly related to quantifying the average duration of immunity 1/δ. In this model extension (Fig. 1 and materials and methods), we incorporate two vaccinated classes; *V*_1_ accounts for individuals who have received one dose of a SARS-CoV-2 vaccine, and *V*_2_ tracks individuals who have received two doses. In the short term, we assume that both dosing options decrease susceptibility by fractions $\left(1-\epsilon_{V_{1}}\right)$ (one dose) and $\left(1-\epsilon_{V_{2}}\right)$ (two doses), inferred from the clinical trial data (although the nature of the infecting variant may influence susceptibility). We also assume that *I_V_* tracks infection after vaccination. We allow for vaccinal immunity to wane at separate rates [ρ_1_ (one dose) and ρ_2_ (two doses)], moving individuals to the partially susceptible immune classes $S_{S_{1}}$ and $S_{S_{2}}$ characterized by (possibly different) levels of immune protection ϵ_1_ and ϵ_2_. Infection after waned one-dose or two-dose vaccinal immunity is tracked by the immune classes $I_{S_{1}}$ and $I_{S_{2}}$, respectively. We consider a continuous spectrum for the interdose period $\left(\frac{1}{\omega }\right)$, with an infinite value corresponding to a one-dose strategy, and model the rate of administration of the first dose ν as an increasing function of the interdose period (Fig. 1 and materials and methods) to reflect the increase in available doses due to a delayed second dose. Thus, dosing regimes with longer interdose periods allow for higher coverage with the first dose.

**Fig. 1:**
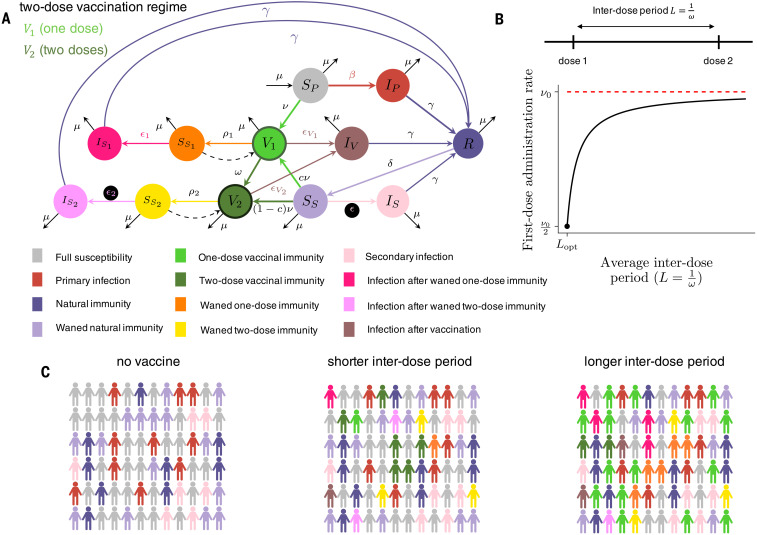
Description of the extended immuno-epidemiological model with one- and two-dose vaccination regimes. Based on (*13*). (**A**) Model flow chart depicting transitions between immune classes (see main text and materials and methods for a full description of the immune classes and parameters). (**B**) Diagram of the interdose period $\left(\frac{1}{\omega }\right)$ between the first and second vaccine doses and its relationship to the rate of administration of the first vaccine dose ν. The maximum achievable rate is ν_0_ for a fully one-dose strategy, and ν is assumed to decrease exponentially to its lowest value ν_0_/2 when a fully two-dose strategy with interdose period corresponding to the clinical recommendation (*L*_opt_) is used. (**C**) Representative schematic of societal composition of various immune classes for the SIR(S) model with no vaccination (left), the extended model with a short interdose period (middle), and the extended model with a long interdose period (right).

We begin by projecting the epidemiological impacts of the different dosing regimes on medium-term temporal dynamics of COVID-19 cases. We then examine the potential evolutionary consequences of each dosing regime by calculating a time-dependent relative net viral adaptation rate (*17*). This term is related to the strength of natural and vaccinal immunity (either via inducing selection through immune pressure or suppressing viral replication) as well as the sizes of classes of individuals experiencing infections after immune waning.

## Epidemiological impacts

As a base case, we consider a high-latitude European or North American city with initial conditions that qualitatively correspond to early 2021 (see supplementary materials and figs. S5 and S6 for other scenarios, e.g., a high initial attack rate or almost full susceptibility), in addition to a seasonal transmission rate (*21*) with NPIs (see materials and methods). Given immunological and future control uncertainties, we are aiming to project qualitatively rather than formulate quantitative predictions for particular locations. The UK and Canadian policy is for a delayed second dose; they are not aiming for an exclusively one-dose policy. However, we explore the one-dose strategy as an extreme case for the two-dose vaccines; this strategy also encompasses a pessimistic situation of waning public confidence in vaccination and individuals’ own decisions to forgo the second dose. This one-dose policy could also capture vaccines that only require a single dose, e.g., the Johnson & Johnson vaccine.

**Fig. 2:**
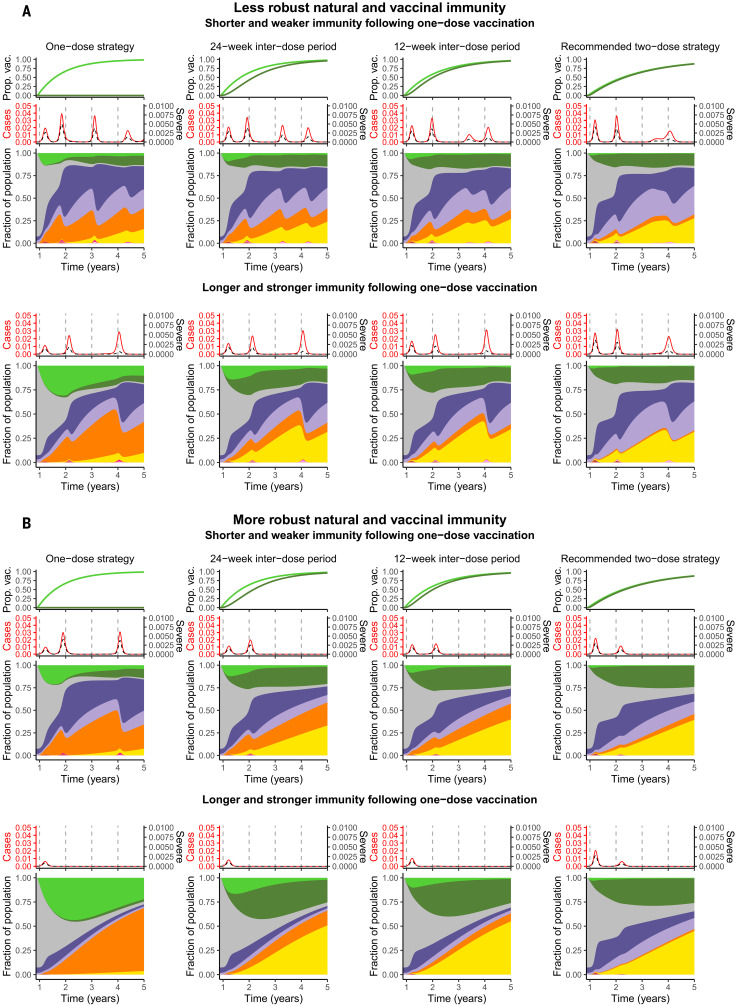
Synoptic medium-term immune landscapes and infection burden. The immune and infection class colors are the same as in Fig. 1A. Each panel shows the following: (Top) Illustrative time series of the fraction of the population vaccinated with one or two doses [see (*56*)]. (Middle) The fraction of total and severe infections [see (*57*)]. (Bottom) Area plots of the fraction of the population that makes up each immune class (*S_P_*, *R*, *S_S_*, *V*_1_, *V*_2_, $S_{S_{1}}$, $S_{S_{2}}$) or infection class (*I_P_*, *I_S_*, *I_V_*, $I_{S_{1}}$, $I_{S_{2}}$) from just before the introduction of vaccination until 5 years after onset of the pandemic. In all plots, the maximum rate of administration of the first vaccine dose is taken to be ν_0_ = 2%, and the vaccine is introduced at *t*_vax_ = 48 weeks. We take $\epsilon_{V_{1}}=0.1$ and $\epsilon_{V_{2}}=0.05$, in keeping with data from clinical trials (*3*). The fraction of severe cases for primary infections, secondary infections, infection after vaccination, and infection after waned two-dose immunity are taken to be $x_{\mathrm{sev},p}=0.14$, $x_{\mathrm{sev},s}=0.07$, $x_{\mathrm{sev},V}=0.14$, and $x_{\mathrm{sev},2}=0$, respectively. The transmission rates and periods of NPI adoption are defined in the materials and methods. The leftmost column corresponds to a one-dose vaccine strategy (ω = 0), followed by interdose spacings of 24 weeks, 12 weeks, and 4 weeks (rightmost column). (**A**) An overall more pessimistic natural and vaccinal immunity scenario, with ϵ = ϵ_2_ = 0.7 and 1/δ = 1/ρ_2_ = 1 year. For a less effective one-dose vaccine (top section), we take ϵ_1_ = 0.9 and 1/ρ_1_ = 0.25 years, and the fraction of severe cases associated with infection after waned one-dose immunity is $x_{\mathrm{sev},1}=0.14$. For an effective one-dose vaccine (bottom section), we take ϵ_1_ = 0.7 and 1/ρ_1_ = 1 year, and the fraction of severe cases associated with infection after waned one-dose immunity is $x_{\mathrm{sev},1}=0$. (**B**) An overall more optimistic natural and vaccinal immunity scenario, with ϵ = ϵ_2_ = 0.5 and 1/δ = 1/ρ_2_ = 2 years. For a less effective one-dose vaccine (top section), we take ϵ_1_ = 0.9 and 1/ρ_1_ = 0.5 years, and the fraction of severe cases associated with infection after waned one-dose immunity is $x_{\mathrm{sev},1}=0.14$. For an effective one-dose vaccine (bottom section), we take ϵ_1_ = 0.5 and 1/ρ_1_ = 2 years, and the fraction of severe cases associated with infection after waned one-dose immunity is $x_{\mathrm{sev},1}=0$.

In Fig. 2, we present potential scenarios for medium-term SARS-CoV-2 infection and immunity dynamics contingent on vaccine dosing regimes. We start by assuming that vaccination occurs at a constant rate, and we also assume a relatively optimistic maximum rate of administration of the first dose of ν_0_ = 2% of the population per week (see supplementary materials for other scenarios). Figure 2A and Fig. 2B correspond, respectively, to scenarios with weaker (and shorter) and stronger (and longer) natural and vaccinal adaptive immune responses. Thus, the former represents a scenario with higher secondary susceptible density than the latter. In each panel, the top and bottom sections consider poor and robust one-dose vaccinal immunity, respectively. The leftmost column represents a one-dose vaccine policy (captured in the model by infinite dose spacing), with dose spacing decreasing to 4 weeks in the rightmost column (i.e., a strict two-dose policy with doses separated by the clinical trial window corresponding to Moderna’s recommendations for their vaccine, hereafter referred to as the “recommended” two-dose strategy).

As expected, we find that broader deployment of widely spaced doses is beneficial. Specifically, a one-dose strategy (or a longer interdose period) may lead to a substantially reduced first epidemic peak of cases after the initiation of vaccination (compare the leftmost top panels of Fig. 2, A and B, with the no-vaccination scenarios in fig. S1, A and B). This result applies even if immunity conferred by one vaccine dose is shorter and weaker than that conferred by two doses (top panels of Fig. 2, A and B). However, under these conditions of imperfect immunity, an exclusively one-dose strategy then leads to an earlier subsequent peak owing to the accumulation of partially susceptible individuals. When the rate of administration of the first dose is very high (fig. S4, ν_0_ = 5% per week), this subsequent infection peak may be larger than that expected in the scenario with no vaccination. In general, the accumulation of partially susceptible individuals with waned one-dose vaccinal immunity can be mitigated by implementing a two-dose strategy and decreasing the time between doses. Thus, in situations of a less effective first dose where the second dose is delayed, it is important to ensure individuals eventually do obtain their second dose.

**Fig. 3:**
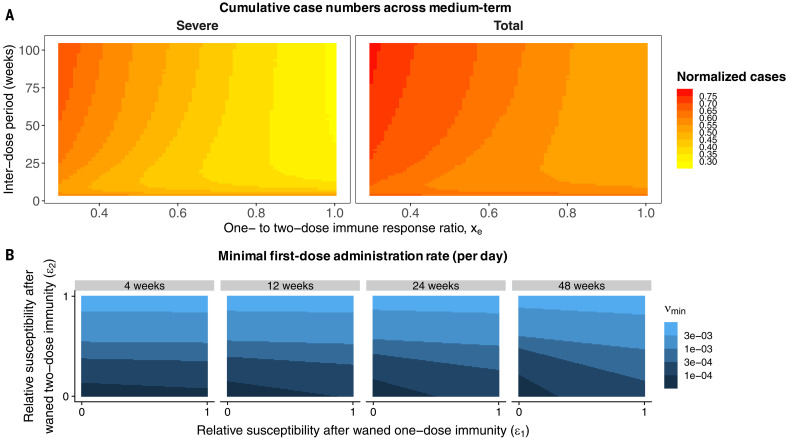
Heatmaps depicting various epidemiological outcomes contingent on dosing regimes. (**A**) Cumulative severe (left) and total (right) case numbers relative to the scenario with no vaccine from the time of vaccine introduction through the end of the 5-year time period after onset of the pandemic as a function of the one-dose to two-dose immune response ratio *x_e_* and the interdose period. Parameters correspond to the weak immunity scenario of Fig. 2A, but *x_e_* sets the value of ϵ_1_, ρ_1_, and $x_{\mathrm{sev},1}$. Specifically, we take ϵ_1_ = ϵ_2_ + (1 − *x_e_*)(1 − ϵ_2_) such that the susceptibility to infection after a waned single dose interpolates linearly between the value after waned two doses (ϵ_2_) when the one- and two-dose immune responses are equally strong (*x_e_* = 1) and unity (full susceptibility) when a single dose offers no immune protection (*x_e_* = 0). Similarly, we take *x*_sev,1_ = *x*_sev,2_ + (1 – *x_e_*)( *x*_sev,_*_V_* – *x*_sev,2_), such that the fraction of severe cases for infections after a waned single dose interpolates linearly between the value after waned two doses (*x*_sev,2_) when *x_e_* = 1 and the value after a (failed) vaccination *x*_sev,_*_V_* when *x_e_* = 0. Finally, ρ_1_ is given by ρ_1_ = ρ_2_/*x_e_*. (**B**) Values of ν_min_, the minimal rate of first dose administration per day such that for any ν > ν_min_ the basic reproduction $R_{0}\left[\nu \right]<1$ and the disease cannot invade (see supplementary materials), as a function of the strength of immunity after one (ϵ_1_) and two (ϵ_2_) waned vaccines doses, for different interdose periods. We take the duration of one-dose and two-dose vaccinal immunity to be 1/ρ_1_ = 0.5 years and 1/ρ_2_ = 1 year, respectively, and set $\epsilon_{V_{1}}=0.1$ and $\epsilon_{V_{2}}=0.05$.

In line with intuition, longer and stronger immunity elicited after a single dose heightens the benefits of a one-dose strategy or of delaying the second dose (compare the top and bottom leftmost panels of Fig. 2, A and B). Additionally, the protective effects of adopting these strategies instead of the two-dose regime are maintained in the medium term, with decreased burden in all future peaks. These effects are further summarized in Fig. 3A via the cumulative number of total and severe cases (right and left panels, respectively) over ~4 years from the time of vaccine initiation, normalized by the burdens predicted when no vaccines are administered; these ratios are plotted as a function of the interdose period and the one- to two-dose immune response ratio *x_e_* (see Fig. 3 caption for details). When the immune response conferred by a single dose is similarly robust to that conferred by two doses, total case numbers (Fig. 3A, right panel) can be substantially reduced by delaying the second dose. However, for smaller values of *x_e_*, larger interdose periods are associated with more cases. The reduction in the cumulative burden of severe cases is even more sizeable (Fig. 3A, left panel) owing to the assumed reduction in the fraction of severe cases for partially immune individuals. When vaccination rates are substantially lower (fig. S2, ν_0_ = 0.1% per week; and fig. S3, ν_0_ = 1% per week), the benefits of a single-dose strategy diminish even for an effective first dose, as an insufficient proportion of the population is immunized. The short-term effect of the vaccine on case numbers is sensitive to when it is introduced in the dynamical cycle (figs. S7 and S8), highlighting the critical interplay between the force of infection and the level of population immunity (see supplementary materials for further details).

Vaccines will be central to efforts to attain community immunity (*22*) and thus prevent local spread due to case importation. We therefore analytically calculated the first vaccine dose administration rate for a given interdose spacing required for community immunity in our model (see supplementary materials). In the long term, however, in countries with adequate supplies, individuals whose one- or two-dose immunity has waned will likely be able to get vaccinated again before reinfection; we therefore incorporated revaccination of these individuals into the extended model and computed an analogous minimal vaccination rate, which we plot in Fig. 3B. We find that as the interdose period grows, this minimal rate depends increasingly on the degree of reduction in susceptibility after the waning of one-dose vaccinal immunity ϵ_1_ (Fig. 3B and see fig. S13 for other parameter choices). Vaccine refusal (*23*) may also affect the attainment of community immunity through vaccinal immunity in the longer term (see supplementary materials).

## Evolutionary impacts

**Fig. 4:**
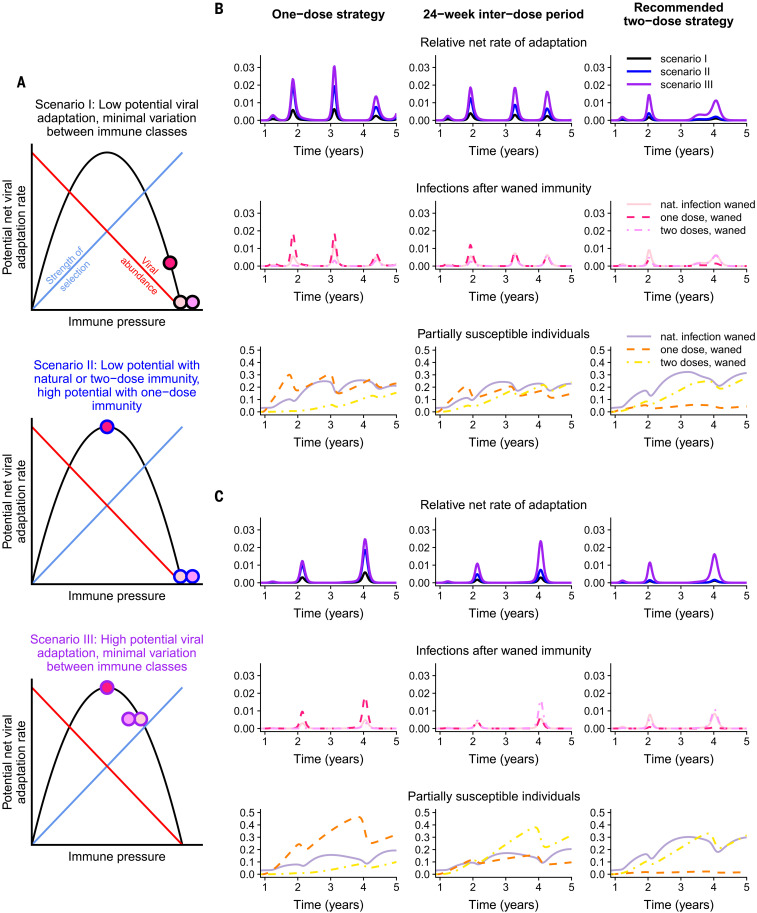
Potential viral evolution scenarios under different vaccine regimes. (**A**) Schematic representations of the potential net viral adaptation rate associated with the *I_S_*, $I_{S_{1}}$, and $I_{S_{2}}$ infection classes under three different scenarios. These are illustrated by the filled circles, with the inside color denoting the infection class and corresponding to the legend in Fig. 1A. The circle borders correspond to the three scenarios considered (scenario I: black, top panel; scenario II: blue, middle panel; and scenario III: purple, bottom panel). The phylodynamic model for potential viral adaptation as a function of immune pressure is adapted from (*17*). (**B** and **C**) Relative net rates of adaptation [top rows; colors correspond to the scenarios in (A)] and composition of associated infection classes (middle rows; *I_S_*, solid lines; $I_{S_{1}}$, dashed lines; $I_{S_{2}}$, dashed-dotted lines) and susceptible classes (bottom rows; *S_S_*, solid lines; $S_{S_{1}}$, dashed lines; $S_{S_{2}}$, dashed-dotted lines). The colors in the middle and bottom rows correspond to the legend in Fig. 1A. The leftmost column corresponds to a one-dose strategy, an interdose period of $\frac{1}{\omega }=24$ weeks is assumed in the middle column, and the rightmost column assumes a two-dose strategy with doses separated by the recommended window of $\frac{1}{\omega }=4$ weeks. Both (B) and (C) correspond to a weak natural and vaccinal immunity scenario, with the same parameters as those in Fig. 2A. A weaker immune response after one vaccine dose is assumed in (B) (with parameters corresponding to those in the top section of Fig. 2A), and a stronger immune response after one vaccine dose is assumed in (C) (with parameters corresponding to those in the bottom section of Fig. 2A). The weights used to calculate the relative net rates of adaptation are $w_{IS,I}=0.05$, $w_{IS_{1},I}=0.3$, and $w_{IS_{2},I}=0.05$ in scenario I; $w_{IS,II}=0.05$, $w_{IS_{1},II}=1$, and $w_{IS_{2},II}=0.05$ in scenario II; and $w_{IS,III}=0.8$, $w_{IS_{1},III}=1$, and $w_{IS_{2},III}=0.8$ in scenario III.

The recent emergence of numerous SARS-CoV-2 variants in still relatively susceptible populations underlines the virus’s evolutionary potential (*24*–*26*). We focus here on the longer-term potential for immune escape from natural or vaccinal immunity (*17*). For immune escape variants to spread within a population, they must first arise via mutation, and then there must be substantial selection pressure in their favor. We expect the greatest opportunity for variants to arise in (and spread from) hosts with the highest viral loads, likely those with the least immunity. On the other hand, we expect the greatest selection for escape where immunity is strongest. Previous research on the phylodynamic interaction between viral epidemiology and evolution (based on seasonal influenza) predicts that partially immune individuals (permitting intermediate levels of selection and transmission) could maximize levels of escape (*17*) (Fig. 4A). Under this model, we would project that different categories of secondarily infected people (after waning of natural immunity or immunity conferred by one or two doses of vaccine) would be key potential contributors to viral immune escape.

In Fig. 4, we consider three potential evolutionary scenarios, exploring different assumptions regarding viral abundance and within-host selection for the various immune classes. In all scenarios, we assume, for simplicity, that immunity elicited after two doses of the vaccine is equivalent to that elicited after natural infection. We also assume that transmission rises with viral abundance in hosts (*17*). In scenario I (black-bordered circles, top panel of Fig. 4A), we assume that infections of all classes of partially susceptible individuals lead to strong selective pressures and low viral abundance (a marker of low transmission) and thus low rates of adaptation, with only slightly reduced immune pressure for infections after a waned single vaccine dose relative to natural infection or two doses. Scenario II (blue-bordered circles, middle panel of Fig. 4A) considers a situation where natural and two-dose vaccinal immunity again lead to low viral abundance but where one-dose vaccinal immunity is associated with intermediate immune pressure that results in substantially higher rates of viral adaptation. Finally, in scenario III (purple-bordered circles, bottom panel of Fig. 4A), adaptive immune responses after waned natural, one-dose, and two-dose vaccinal immunity all lead to similar intermediate levels of immune pressure and high rates of viral adaptation. In all cases, we assume, for tractability, that viral immune escape is not correlated with clinical severity (*27*).

The relative potential viral adaptation rates [see (*17*) for more details] corresponding to each scenario are presented in the top rows of Fig. 4, B and C. This relative rate is estimated as the sum of the sizes of the infection classes after waned immunity (i.e., *I_S_* after *S_S_*, $I_{S_{1}}$ after $S_{S_{1}}$, and $I_{S_{2}}$ after $S_{S_{2}}$) weighted by the infection class–specific net viral adaptation rate assigned in each scenario. Therefore, this quantity reflects a weight-averaged potential rate for viral adaptation per individual per infection. The corresponding immune and susceptibility classes are plotted in the middle and bottom rows, respectively, according to the color scheme defined in Fig. 1A. The weaker immunity scenario of Fig. 2A is considered, with Fig. 4, B and C, corresponding, respectively, to the situations of a weaker and more robust single vaccine dose relative to two doses. The leftmost column corresponds to a one-dose strategy, an interdose period of $\frac{1}{\omega }=24$ weeks is assumed in the middle column, and the rightmost column assumes a two-dose strategy with doses separated by the clinical trial window of $\frac{1}{\omega }=4$ weeks.

Different assumptions regarding the strength and duration of adaptive immune responses to vaccines and natural infections alter projections for the proportions of individuals in the partially susceptible immune classes over time. When one-dose vaccinal immunity is poor, a one-dose strategy results in the rapid accumulation of partially susceptible $S_{S_{1}}$ individuals (Fig. 4B, bottom row) and a greater infection burden. (Note, this $S_{S_{1}}$ immune class is highlighted in orange for visibility in Figs. 1, 2, and 4.) When the assumed individual rates of evolutionary adaptation arising from these infection classes are high (scenarios II and III), we find that a one-dose strategy could lead to substantially higher relative rates of adaptation. This effect can be mitigated by implementing a two-dose strategy even with a longer interdose period than the recommended duration, echoing our epidemiological findings.

A single-dose strategy of a strongly immunizing vaccine reduces infection rates, resulting in lower relative rates of adaptation when a one-dose strategy is used; however the resulting large fraction of $S_{S_{1}}$ individuals may still lead to evolutionary pressure, particularly when the potential viral adaptation rate associated with $I_{S_{1}}$ infections is large. A two-dose strategy mitigates this effect, but the corresponding reduction in vaccinated individuals increases the infection burden from other classes. Thus, our results highlight the importance of rapid vaccine deployment to avoid these potentially pessimistic evolutionary outcomes. More broadly, our results further underline the importance of equitable, global vaccination (*28*, *29*): Immune escape anywhere will quickly spread.

## Impact of increasing vaccination over time

In the supplementary materials (figs. S10 to S12), we explore the implications of ramping up vaccine deployment through two approaches. First, we examine a simple increase in the rate of administration of the first dose and unchanged dosing regimes (fig. S10). Qualitatively, these results are largely analogous to our previous results and reflect the benefits of increasing population immunity through an increase in vaccination deployment.

However, as vaccines become more widely available, policies on dosing regimes may change. The second approach we consider is a timely shift to a two-dose policy with recommended interdose spacing as vaccine deployment capacity increases (figs. S11 and S12). Initially delaying (or omitting) the second dose decreases the first epidemic peak after the initiation of vaccination. Such a reduction in first peak size would also reduce secondary infections and thus potentially immune escape in most cases (i.e., an evolutionary advantage). Subsequently, the switch to a manufacturer-timed vaccine dosage regime mitigates the potential medium-term disadvantages of delaying (or omitting) the second dose that may arise if immunity conferred from a single dose is relatively poor, including the accumulation of partially susceptible $S_{S_{1}}$ individuals whose one-dose vaccinal immunity has waned. These contrasts highlight the importance of data-driven policies that undergo constant reevaluation as vaccination progresses.

## Caveats

Our immuno-epidemiological model makes several assumptions. Although heterogeneities (superspreading, age, space, etc.) (*30*–*33*) are important for the quantitative prediction of SARS-CoV-2 dynamics, we previously found that these do not qualitatively affect our results (*13*). Nevertheless, we again briefly explore the epidemiological consequences of heterogeneities in transmission and vaccine coverage in the supplementary materials. We have also assumed that the robustness of immune responses after the second dose is independent of the interdose period, yet it is possible that delaying the second dose may actually enhance adaptive immune responses (*34*). Detailed clinical evaluation of adaptive immune responses after one and two vaccine doses with different interdose spacing is an important direction for future work.

Additionally, we have assumed highly simplified scenarios for NPIs. The chosen scenario was selected to qualitatively capture current estimates of SARS-CoV-2 prevalence and seropositivity in large cities. However, these values vary substantially between locations, a notable example being recent estimates of a large infection rate in Manaus, Brazil, during the first wave (*35*) or countries having almost no infections, owing to the successful implementation of NPIs (*36*–*38*). We have examined these scenarios in the supplementary materials (figs. S5 and S6). The qualitative projections of our model are sensitive to the composition of infection and immune classes at the onset of vaccination (including, therefore, the assumption of drastically higher seropositivity levels, i.e., the sum of the *S_S_* and *R* classes). We further explore this in the supplementary materials through the initiation of vaccination at different times in the dynamic cycle (figs. S7 and S8). Thorough explorations of various NPIs, seasonal transmission rate patterns, vaccine deployment rates, dosing regimes, and clinical burdens can be investigated for broad ranges of epidemiological and immunological parameters with the online interactive application (*39*).

Finally, we have explored the simplest evolutionary model, which can only give a general indication of the potential for evolution under different scenarios. Including more-complex evolutionary models (*40*, *41*) into our framework is thus another important area for future work. Population heterogeneities likely have complex impacts on viral evolution. First, heterogeneities in immune responses and transmission [e.g., chronically infected hosts that shed virus for extended periods (*42*), or focused versus polyclonal responses] may have important impacts on the accumulation of genetic diversity and the strength of selection pressures and hence on evolutionary potential [e.g., for influenza, see (*43*)]. Second, there are complex evolutionary implications of disease severity minimization by vaccination (*27*, *44*). Third, superspreading and contact structure could influence the rate of spread of novel variants through a population (*45*). Additionally, increases in viral avidity to the human angiotensin-converting enzyme 2 receptor might generate multiple benefits for the virus in terms of enhanced transmission and immune escape (*46*). Finally, genetic processes such as clonal interference, epistasis, and recombination also add substantial complexity to evolutionary dynamics [e.g., (*17*, *47*, *48*)]. Further model refinements should also include these details for increased accuracy. A full list of caveats is presented in the supplementary materials.

## Conclusion

The deployment of SARS-CoV-2 vaccines in the coming months will strongly shape postpandemic epidemiological trajectories and characteristics of accumulated population immunity. Dosing regimes should seek to navigate existing immunological and epidemiological trade-offs between individuals and populations. Using simple models, we have shown that different regimes may have crucial epidemiological and evolutionary impacts, resulting in a wide range of potential outcomes in the medium term. Our work also lays the foundation for a number of future considerations related to vaccine deployment during ongoing epidemics, especially preparing against future pandemics.

In line with intuition, spreading single doses in emergency settings (i.e., rising infections) is beneficial in the short term and reduces prevalence. Furthermore, we find that if immunity after a single dose is robust, then delaying the second dose is also optimal from an epidemiological perspective in the longer term. On the other hand, if one-dose vaccinal immunity is weak, the outcome could be more pessimistic; specifically, a vaccine strategy with a very long interdose period could lead to marginal short-term benefits (a decrease in the short-term burden) at the cost of a higher infection burden in the long term and substantially more potential for viral evolution. These negative longer-term effects may be alleviated by the eventual administration of a second dose, even if it is moderately delayed. With additional knowledge of the relative strength and duration of one-dose vaccinal immunity and corresponding clinically informed policies related to dosing regimes, pessimistic scenarios may be avoided. For context, at the time of writing, the UK, for example, has been particularly successful in rolling out vaccination to a large population with a wide spacing between doses (*49*). Our model illustrates that, ultimately, the long-term impacts of this strategy, especially in terms of transmission and immune escape, will depend on the duration and strength of one-dose vaccinal immunity. Recent evidence of weaker vaccinal immunity against the B.1.351 strain (*50*) underlines the importance of both detecting novel strains and titrating the strength of natural and vaccinal immunity against them.

Our results stress the negative epidemiological and evolutionary impacts that may emerge in places where vaccine deployment is delayed and vaccination rates are low. And because these consequences (e.g., the evolution of new variants) could emerge as global problems, this underlines the urgent need for global equity in vaccine distribution and deployment (*28*, *29*).

Current uncertainties surrounding the strength and duration of adaptive immunity in response to natural infection or vaccination lead to very broad ranges for the possible outcomes of various dosing regimes. Nevertheless, ongoing elevated COVID-19 case numbers stress the urgent need for effective mass vaccine deployment. Overall, our work emphasizes that the impact of vaccine dosing regimes is strongly dependent on the relative robustness of immunity conferred by a single dose. It is therefore imperative to determine the strength and duration of clinical protection and transmission-blocking immunity through careful clinical evaluations (including, for instance, randomized control trials of dose intervals and regular testing of viral loads in vaccinated individuals, their contacts, and those who have recovered from natural infections) in order to enforce sound public policies. More broadly, our results underscore the importance of exploring the phylodynamic interaction of pathogen dynamics and evolution, from within-host to global scales, for SARS-CoV-2, influenza, and other important pathogens (*40*, *41*, *47*, *48*, *51*, *52*).

## Materials and methods

### Model formulation

We extend the model of (*13*) to examine different vaccination strategies. The additional compartments are as follows: *V_i_* denotes individuals vaccinated with *i* doses and are thus immune; $S_{S_{i}}$ denotes individuals whose complete *i*-dose immunity has waned and are now partially susceptible again; $I_{S_{i}}$ denotes individuals who were in $S_{S_{i}}$ and have now been infected again; *I_V_* denotes individuals for whom the vaccine did not prevent infection.

The extended model contains several new parameters: $\frac{1}{\rho_{i}}$ is the average duration of vaccinal immunity *V_i_*; $\frac{1}{\omega }$ is the average interdose period; $\epsilon_{V_{i}}$ is the decrease in susceptibility after vaccination with dose *i*; ϵ*_i_* is the (decreased) susceptibility after waning of *i*-dose immunity; α*_i_* is the relative infectiousness of individuals in $I_{S_{i}}$; and α*_V_* is the relative infectiousness of individuals in *I_V_*. To allow for heterogeneity in vaccinal immune responses and potentially cumulative effects of natural and vaccinal immunity, we take *c* to be the fraction of previously infected partially susceptible individuals (*S_S_*) for whom one dose of the vaccine gives equivalent immunity to two doses for fully susceptible individuals (*S_P_*). Finally, *x_i_* is the fraction of individuals in $S_{S_{i}}$ that are revaccinated, and (1 − *p_i_*) is the fraction of individuals in $S_{S_{i}}$ for whom readministration of the first dose provides equivalent immune protection to two doses (i.e., they transition to the *V*_2_ class). The full set of equations governing the transitions between these infection and immunity classes is then given by

$$\frac{dS_{P}}{dt}=\;\mu -\;\beta S_{P}\left[I_{P}+\alpha I_{S}+\alpha_{V}I_{V}+\alpha_{1}I_{S_{1}}+\alpha_{2}I_{S_{2}}\right]-\left(s_{\mathrm{vax}}\nu +\mu \right)S_{P}$$ (1A)

$$\frac{dI_{P}}{dt}=\;\beta S_{P}\left[I_{P}+\alpha I_{S}+\alpha_{V}I_{V}+\alpha_{1}I_{S_{1}}+\alpha_{2}I_{S_{2}}\right]-\left(\gamma +\mu \right)I_{P}$$ (1B)

$$\frac{dR}{dt}=\gamma \left(I_{P}+I_{s}+I_{V}+I_{S_{1}}+I_{S_{2}}\right)-\left(\delta +\mu \right)R$$ (1C)

$$\frac{dS_{S}}{dt}=\;\delta R-\;\epsilon \beta S_{S}\left[I_{P}+\alpha I_{S}+\alpha_{V}I_{V}+\alpha_{1}I_{S_{1}}+\alpha_{2}I_{S_{2}}\right]-\left(s_{\mathrm{vax}}\nu +\mu \right)S_{S}$$ (1D)

$$\frac{dI_{S}}{dt}=\;\epsilon \beta S_{S}\left[I_{P}+\alpha I_{S}+\alpha_{V}I_{V}+\alpha_{1}I_{S_{1}}+\alpha_{2}I_{S_{2}}\right]-\left(\gamma +\mu \right)I_{S}$$ (1E)

$$\begin{array}{l} \frac{dV_{1}}{dt}=s_{\mathrm{vax}}\nu S_{P}+cs_{\mathrm{vax}}\nu S_{S}+x_{1}p_{1}s_{\mathrm{vax}}\nu S_{S_{1}}+x_{2}p_{2}s_{\mathrm{vax}}\nu S_{S_{2}}-\\ \epsilon_{V_{1}}\beta V_{1}\left[I_{P}+\alpha I_{S}+\alpha_{V}I_{V}+\alpha_{1}I_{S_{1}}+\alpha_{2}I_{S_{2}}\right]-\left(\omega +\rho_{1}+\;\mu \right)V_{1} \end{array}$$ (1F)

$$\begin{array}{l} \frac{dV_{2}}{dt}=\left(1-c\right)s_{\mathrm{vax}}\nu S_{S}+x_{1}\left(1-p_{1}\right)s_{\mathrm{vax}}\nu S_{S_{1}}+x_{2}\left(1-p_{2}\right)s_{\mathrm{vax}}\nu S_{S_{2}}+\omega V_{1}-\\ \epsilon_{V_{2}}\beta V_{2}\left[I_{P}+\alpha I_{S}+\alpha_{V}I_{V}+\alpha_{1}I_{S_{1}}+\alpha_{2}I_{S_{2}}\right]-\left(\rho_{2}+\;\mu \right)V_{2} \end{array}$$ (1G)

$$\frac{dI_{V}}{dt}=\beta \left(\epsilon_{V_{1}}V_{1}+\epsilon_{V_{2}}V_{2}\right)\left[I_{P}+\alpha I_{S}+\alpha_{V}I_{V}+\alpha_{1}I_{S_{1}}+\alpha_{2}I_{S_{2}}\right]-\left(\gamma +\mu \right)I_{V}$$ (1H)

$$\frac{dS_{S_{1}}}{dt}=\rho_{1}V_{1}-\epsilon_{1}\beta S_{S_{1}}\left[I_{P}+\alpha I_{S}+\alpha_{V}I_{V}+\alpha_{1}I_{S_{1}}+\alpha_{2}I_{S_{2}}\right]-\left(s_{\mathrm{vax}}x_{1}\nu +\mu \right)S_{S_{1}}$$ (1I)

$$\frac{dS_{S_{2}}}{dt}=\rho_{2}V_{2}-\epsilon_{2}\beta S_{S_{2}}\left[I_{P}+\alpha I_{S}+\alpha_{V}I_{V}+\alpha_{1}I_{S_{1}}+\alpha_{2}I_{S_{2}}\right]-\left(s_{\mathrm{vax}}x_{2}\nu +\mu \right)S_{S_{2}}$$ (1J)

$$\frac{dI_{S_{1}}}{dt}=\epsilon_{1}\beta S_{S_{1}}\left[I_{P}+\alpha I_{S}+\alpha_{V}I_{V}+\alpha_{1}I_{S_{1}}+\alpha_{2}I_{S_{2}}\right]-\left(\gamma +\mu \right)I_{S_{1}}$$ (1K)

$$\frac{dI_{S_{2}}}{dt}=\epsilon_{2}\beta S_{S_{2}}\left[I_{P}+\alpha I_{S}+\alpha_{V}I_{V}+\alpha_{1}I_{S_{1}}+\alpha_{2}I_{S_{2}}\right]-\left(\gamma +\mu \right)I_{S_{2}}$$ (1L)

For all simulations, we take μ = 0.02 year^−1^ corresponding to a yearly crude birth rate of 20 per 1000 people. Additionally, we take the infectious period to be 1/γ = 5 days, consistent with the modeling in (*13*, *21*, *53*) and the estimation of a serial interval of 5.1 days for COVID-19 in (*54*) and assume that *c* = 0.5. We take the relative transmissibility of infections to be α = α*_V_ =* α_1_ *=* α_2_ = 1, and therefore only modulate the relative susceptibility to disease ϵ. For the initial conditions of all simulations, we take *I_P_* = 1 × 10^−9^ and assume the remainder of the population is in the fully susceptible class. The values of the remaining parameters used in the various simulations are specified throughout the main text.

### Determination of seasonal reproduction numbers

To reflect observed seasonal variation in transmission rates for respiratory infections arising from related coronaviruses (*21*), influenza (*21*), and respiratory syncytial virus (*55*), we base seasonal reproduction numbers in this work on those in (*13*), which were calculated in (*21*) on the basis of the climate of New York City. Other seasonal patterns can be explored using the interactive online application (*39*). In all simulations, we modify these values to force a mean value for the basic reproduction number of ${\bar{R}}_{0}=\langle R_{0}\left(t\right)\rangle =2.3$ by multiplying the climate-derived time series *R*_0,_*_c_*(*t*) by 2.3 and dividing by its average value

$$R_{0}\left(t\right)=R_{0,c}\left(t\right)\frac{2.3}{{\bar{R}}_{0,c}}$$

### Modeling of nonpharmaceutical interventions (NPIs)

In all simulations, we enforce periods of NPI adoption (arising from behaviors and policies such as lockdowns, mask-wearing, and social distancing) in which the transmission rate is reduced from its seasonal value described in the previous section. In particular, we assume that NPIs are adopted between weeks 8 and 47 after pandemic onset, resulting in the transmission rate being reduced to 45% of its seasonal value. Between weeks 48 and 79, we assume that the transmission rate is 30% higher than for the previous time interval [reflecting an overall reduction to 45 × 1.3 = 58.5% of the original transmission rate], due to either behavioral changes after the introduction of the vaccine or the emergence of more-transmissible strains. Finally, we assume that NPIs are completely relaxed beyond week 80.

### Linking vaccination rate to interdose period

We consider an exponential relationship between the rate of administration of the first vaccination dose ν[ω] and the interdose period $\frac{1}{\omega }$. We assume that this rate is maximized at ν_0_ when no second dose occurs (i.e., ω = 0, an infinite interdose period) and that when the first and second doses are spaced by the clinically recommended interdose period *L*_opt_ $\left(\omega_{\mathrm{opt}}=\frac{1}{L_{\mathrm{opt}}}\right)$, the rate of administration of the first dose is one-half of its maximum value. Thus, $\nu \left[\omega \right]=2^{-L_{\mathrm{opt}}\omega }\nu_{0}$.

## Supplementary Materials

science.sciencemag.org/content/372/6540/363/suppl/DC1 Supplementary Text Figs. S1 to S14 References (*59*–*64*) MDAR Reproducibility Checklist

## Appendix

View/request a protocol for this paper from *Bio-protocol*.
